# Second allogeneic transplants in children: twelve years of experience

**DOI:** 10.7705/biomedica.7946

**Published:** 2026-03-02

**Authors:** Alexis Antonio Franco, Eliana Manzi, Sergio Cabrera-Salcedo, Diana Muñoz-Caluce, Diego Medina

**Affiliations:** 1 Unidad de Trasplante de Médula Ósea, Servicio de Hemato-Oncología Pediátrica, Departamento Materno-Infantil, Fundación Valle del Lili, Cali, Colombia Fundación Valle del Lili Cali Colombia; 2 Facultad de Ciencias de la Salud, Universidad Icesi, Cali, Colombia Universidad Icesi Facultad de Ciencias de la Salud Universidad Icesi Cali Colombia; 3 Centro de Investigaciones Clínicas, Fundación Valle del Lili, Cali, Colombia Fundación Valle del Lili Cali Colombia

**Keywords:** bone marrow transplant, pediatrics, recurrence, immunosuppression, graft vs host disease, trasplante de medula ósea, pediatría, recurrencia, inmunosupresión, enfermedad de injerto contra huésped

## Abstract

**Introduction.:**

A second hematopoietic stem cell transplant is required when the first transplant fails, usually due to relapse or graft failure, and is associated with increased morbidity and mortality. Survival rates range from 74 to 82% in non-neoplastic diseases and from 39 to 58% in neoplastic conditions. Evidence on second hematopoietic stem cell transplant in children is limited, particularly in low- and middle-income countries.

**Objective.:**

To describe the clinical characteristics, complications, and outcomes of children who underwent a second transplant at a high-complexity center between 2012 and 2024.

**Materials and methods.:**

Case series study with descriptive and survival analysis using the Kaplan-Meier method in Stata 14™.

**Results.:**

A total of 346 allogeneic transplants were performed, of which 20 patients underwent a second transplant. Of these, 17 received a haploidentical donor for their second transplant, and the primary indication was a neoplastic disease in 13 cases. The second transplant was performed due to graft failure in 11 of the 20 patients, and due to relapse in the remaining 9. The mean age was 10.7 ± 5 years, with a male predominance (14 out of 20). Haploidentical transplants accounted for 16 of the 20 cases, and 11 used the same donor. The most common complications were acute graft-versus-host disease in 7 cases, 2 of grade III, cytomegalovirus infection in 10 cases, and graft failure after the second transplant in 4 cases: 3 primary, 1 secondary. Transplant-related mortality was 31%. The 2-year overall survival was 54%, with a median follow-up of 11 months.

**Conclusions.:**

Second hematopoietic stem cell transplant is a viable therapeutic option when no other alternatives are available, particularly in resource-limited settings.

## Resumen

**Introducción.:**

El segundo trasplante de progenitores hematopoyéticos es un procedimiento de alto riesgo, indicado principalmente por falla del injerto o recaída de la enfermedad. La supervivencia global varía entre el 40 y el 82 % en las enfermedades no neoplásicas y entre el 39 y el 58 % en las neoplásicas. La evidencia en la población pediátrica es limitada, especialmente en los países de ingresos bajos y medianos.

**Objetivo.:**

Describir las características clínicas, complicaciones y desenlaces en niños que recibieron un segundo trasplante en un centro de alta complejidad entre el 2012 y el 2024.

**Materiales y métodos.:**

Se llevó a cabo un estudio de serie de casos con análisis descriptivo y de supervivencia mediante el método de Kaplan-Meier, utilizando el *software* Stata 14™.

**Resultados.:**

De 410 trasplantes practicados, 346 fueron alogénicos; 20 pacientes recibieron un segundo trasplante alogénico. La mayoría (13/20) presentaba enfermedad neoplásica. Diecisiete de estos trasplantes fueron haploidénticos. Las indicaciones del segundo trasplante fueron por falla del injerto (11/20) y recaída (9/20). La edad media fue de 10 ± 5 años; 14 eran varones. Dieciséis segundos trasplantes fueron haploidénticos, y en 11 se utilizó el mismo donante. Las complicaciones frecuentes incluyeron la enfermedad aguda de injerto contra huésped (7 pacientes, 2 con grado III), la infección por citomegalovirus (10 pacientes) y la falla de injerto posterior al trasplante (4 casos). La mortalidad relacionada fue del 31 %. La supervivencia global a 2 años fue del 54 %, con una mediana de seguimiento de 11 meses.

**Conclusiones.:**

El segundo trasplante de progenitores hematopoyéticos puede ser una alternativa viable en niños cuando no existen otras opciones terapéuticas, especialmente en contextos con recursos limitados.

Allogeneic hematopoietic stem cell transplantation is a curative option for several hematologic diseases, both neoplastic and non-neoplastic, in the pediatric population ^1^. However, post-transplant relapses, graft failure, either primary or secondary, remain significant clinical challenges, leading to the consideration of a second pediatric allogeneic hematopoietic stem cell transplantation in selected cases ^1^^-^^3^. In most cases, a second allogeneic transplant is the only available rescue option when needed ^4^.

Survival rates after a second allogeneic transplant vary considerably, ranging from 20 to 82%, depending on the population studied. Higher survival rates have been reported in non-neoplastic diseases compared to neoplastic conditions ^2^. Prognostic factors described include time to relapse, type of graft failure, conditioning regimen, and donor selection ^5^. Most studies have been conducted in high-income countries, and there is no consensus on the optimal strategy for a second allogeneic transplant. Various conditioning regimens, generally of reduced intensity, have been used, with either the same or a different donor, including haploidentical donors, with favorable outcomes reported ^6^.

The objective of this study was to describe the clinical characteristics, complications, and outcomes of patients under 18 years of age who underwent a second allogeneic transplant at a high-complexity healthcare center between 2012 and 2024.

## Materials and methods

This study is a case series of patients under 18 years old diagnosed with hematologic malignancies or other non-malignant disorders, such as aplastic anemia, hemoglobinopathies, and immunodeficiencies, who underwent a second pediatric allogeneic hematopoietic stem cell transplantation at Fundación Valle del Lili between 2012 and 2024.

For the first transplantation, recorded variables included the patient's age at the time of the procedure, the transplant primary indication, the type of donor, and the graft source, whether bone marrow or peripheral blood stem cells.

Regarding the second transplantation, pre-transplant data included: transplant date, indication for the procedure, type of graft failure, patient age, conditioning regimen intensity, graft source, donor selection, and graft-versus-host disease prophylaxis protocol.

Post-transplant variables included time to neutrophil recovery, incidence of relapse, primary or secondary graft failure, and post-transplant complications such as acute graft-versus-host disease, cytomegalovirus infection, and hemorrhagic cystitis. Data were collected from electronic medical records and recorded in the REDCap electronic data capture system.

### Statistical analysis

A descriptive statistical analysis was performed, expressing categorical variables as proportions and continuous variables as medians with interquartile ranges. Outcomes were assessed two years after the second transplantation, and the Kaplan-Meier method was used for overall survival analysis from the date of the second transplantation to death from any cause or censoring at the date of the last follow-up during the study period. The second analyzed event was transplant-related mortality from the date of the second transplantation to day +100 or censoring in cases of disease relapse, at the date of relapse diagnosis. The analysis was performed using Stata 14™ software.

## Results

Between 2012 and 2024, a total of 410 hematopoietic stem cell transplants were performed, of which 346 were allogeneic transplants. Of them, 20 patients required a second transplant due to disease relapses or graft failure.

The demographic and clinical characteristics of patients undergoing a second transplantation are presented in table 1. The mean age at the time of the second transplant was 10.7 ± 5 years, with a predominance of male patients (14 of the 20 cases). The underlying disease for the first hematopoietic stem cell transplantation was a malignant disease in 13 patients, while 7 patients underwent transplantation for non-malignant conditions.

**Table 1:** Characteristics of patients with second allogeneic hematopoietic stem cell transplantation

ID	Age (years)	Sex Underlying disease		Cause of second allo-HSCT	Time between HSCT’s (months)	Myeloablative conditioning at first HSCT	Donor/source at first HSCT	Myeloablative conditioning at second allo-HSCT	Donor/source at second allo-HSCT	aGVHD grade III-IV of second allo-HSCT	Death/cause of death	Time to event (months)
1	19	M	PID	Graft failure	2.4	Yes	Haplo/PBSC	Yes	Haplo/PBSC	No	No	-
2	14	M	BMF	Graft failure	6.7	Yes	Haplo/BM	No	Haplo/PBSC	No	Yes/Infection	2
3	17	M	HL	Relapse	11.3	Yes	Autologous/PBSC	No	Haplo/BM	No	No	-
4	9	M	AML	Relapse	21.9	Yes	MSD/PBSC	No	MSD/PBSC	No	No	-
5	15	M	AML	Graft failure	1.8	Yes	Haplo/BM	Yes	Haplo/BM	No	Yes/Graft failure	1.1
6	8	M	AML	Graft failure	4.1	No	MSD/PBSC	No	Haplo/PBSC	No	No	-
7	10	F	ALL	Graft failure	7.6	Yes	Haplo/PBSC	No	Haplo/PBSC	No	Yes/Graft failure	1.3
8	1	M	PID	Graft failure	4.9	Yes	MSD/BM	Yes	MSD/BM	No	No	-
9	17	M	AML	Relapse	Missing	Missing	Missing /PBSC	Yes	MSD/BM	No	No	-
10	15	M	ALL	Relapse	5.9	No	Haplo/PBSC	No	Haplo/PBSC	No	Yes/Graft failure	0.9
11	12	F	ALL	Graft failure	1.5	Yes	Haplo/PBSC	Yes	Haplo/PBSC	Yes	Yes/Graft failure	2
12	6	M	PID	Graft failure	15.6	No	MSD/BM	No	MSD/BM	No	Yes/Graft failure	0.7
13	8	F	AML	Relapse	40.9	No	Haplo/PBSC	Yes	Haplo/PBSC	No	No	-
14	5	F	BMF	Relapse	34.9	Yes	Haplo/BM	Yes	Haplo/BM	No	No	-
15	7	M	AML	Relapse	9.7	Yes	Haplo/BM	Yes	Haplo/BM	No	Yes/Relapse	33.8
16	11	M	ALL	Relapse	50.1	Yes	Haplo/BM	Yes	Haplo/PBSC	No	No	-
17	0.6	F	BMF	Graft failure	1.6	Missing	Missing/BM	No	Haplo/BM	No	Yes/ARDS	12.7
18	2	M	AML	Relapse	3.6	No	Haplo/PBSC	Yes	Haplo/PBSC	No	Yes/Relapse	2.8
19	15	M	BMF	Graft failure	2.0	No	Haplo/BM	No	Haplo/PBSC	No	Yes/Infection	4.6
20	14	F	HBP	Graft failure	2.1	No	Haplo/BM	No	Haplo/BM	Yes	No	-

aGVHD: acute graft-versus-host disease; ALL: acute lymphoblastic leukemia; AML: acute myeloid leukemia; ARDS: acute respiratory distress syndrome; BMF: bone marrow failure syndromes; BM: bone marrow; F: female; HBP: hemoglobinopathies; HL: Hodgkin lymphoma; HSCT: hematopoietic stem cells transplantation; M: male; MSD: matched sibling donor; PBSC: peripheral blood stem cells; PID: primary immunodeficiency diseases

Regarding the characteristics of the first transplant, 13 haploidentical donors were used. The graft source in this procedure was bone marrow in 7 of 13 cases and peripheral blood stem cells in 6 of 13 cases. The median time between the first and second transplantation was 5.9 months (interquartile range: 2.1 to 15).

The second transplantation was mainly performed due to graft failure in 11 of 20 patients, while 9 underwent transplantation due to disease relapse. Most patients received a transplant from a haploidentical donor (16 of 20), with the same donor used in 11 of the 20 cases. Regarding the conditioning regimen, 10 patients received a myeloablative regimen, while 10 received a reduced-intensity regimen. The graft source in the second procedure was bone marrow in 9 of the 20 cases and peripheral blood stem cells in 11 cases.

### Post-transplant complications

Acute graft-versus-host disease occurred in 7 patients, with grade III-IV involvement in 2 cases. Cytomegalovirus infection was reported in 10 patients, and hemorrhagic cystitis was documented in 5 patients.

Graft failure following the second transplant was identified in 4 patients, with 3 experiencing primary failure and one developing secondary failure.

The median follow-up after the second transplant was 11 months (interquartile range: 2 to 73).

### Survival and transplant-related mortality

**Figure 1:**
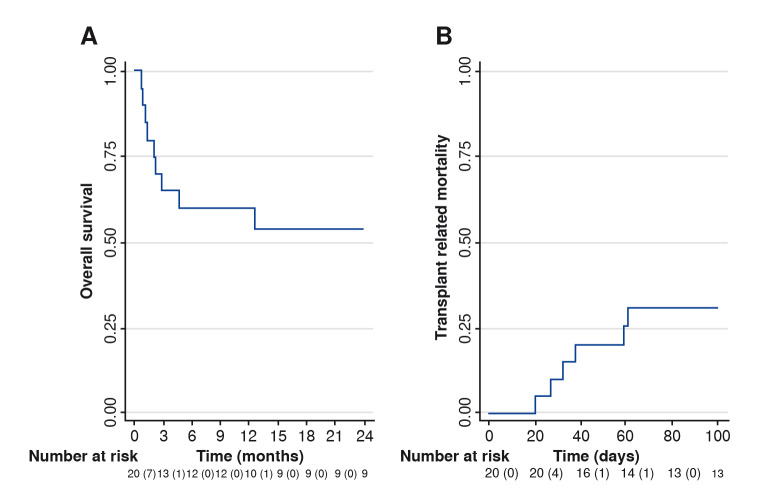
A. Overall survival at two year post-hematopoietic stem cell transplantation. B. Transplant-related mortality at day 100 after second allogeneic hematopoietic stem cell transplantation.

The two-year overall survival was 54% (95% CI: 30 to 73) (figure 1A). The 100-day transplant-related mortality was 31% (95% CI: 15-56) (figure 1B). Causes of death included disease relapse (n=2), graft failure (n=5), bacterial infection (n=2), and acute respiratory distress syndrome (n=1).

## Discussion

In this pediatric series of second allogeneic hematopoietic stem cell transplantation, the two-year overall survival was 54%, within the range reported in previous studies (30-58%) ^7^^-^^9^. Overall survival in malignant diseases was 62%, while in non-malignant diseases it was 43%. This result was not statistically significant, and it falls within the range reported in different populations: 20-40% for neoplastic diseases and 40-85% for nonneoplastic diseases ^9^^-^^14^. However, survival rates described in the literature show wide variability, likely due to differences in the characteristics of the studied populations.

Graft failure is a severe complication with a mortality rate approaching 100% in the absence of rescue therapy, making it one of the main indications for a second transplant ^15^. However, early re-transplantation may increase cumulative toxicity, making the selection of the conditioning regimen a key factor in patient outcomes ^16^. Current evidence supports the second transplantation as the only viable curative option, with outcomes varying according to donor type and conditioning protocol used ^17^.

The incidence of acute graft-versus-host disease was 35% (7/20), including two cases of grade III. These rates are comparable to previous studies, where the incidence of this acute disease in second transplantation ranges from 11.5% to 52%, while chronic graft-versus-host disease occurs in 29-43% of cases ^6^^,^^18^^-^^20^. The incidence of this disease in this series was comparable to that reported after a first transplant. This outcome likely reflects the use of reduced-intensity conditioning combined with antithymocyte globulin and post-transplant cyclophosphamide, a regimen shown to be effective for graft-versus-host disease prevention in both first and second transplants ^21^^-^^23^.

Among post-transplant complications, a high frequency of cytomegalovirus viremia was identified. These findings align with previous reports, where cytomegalovirus infection rates in transplants reach up to 41% ^24^. Viral infections are a major cause of morbidity in this setting and are associated with a significant increase in hospitalization and healthcare costs ^25^. However, in our cohort, no patients died from this disease.

Mortality in our cohort was primarily due to bacterial infections caused by multidrug-resistant pathogens, consistent with other series in which infections are the leading cause of death in hematopoietic stem cell transplantation patients ^7^^,^^26^^,^^27^. Early identification and timely management of these events remain critical challenges in this population, underscoring the need for targeted strategies to prevent and treat post-transplant infectious complications ^28^^,^^29^.

Second pediatric allogeneic hematopoietic stem cell transplantation is a viable strategy, with survival rates aligned with previously reported data and no significant differences between malignant and non-malignant diseases. However, graft failure and infections remain major challenges. In middle-income countries, it represents a therapeutic option for selected patients, highlighting its value in settings similar to ours.

This study has limitations related to the small sample size within clinical subgroups, which restricts the generalizability of the findings. Nevertheless, it provides valuable evidence on second allogeneic hematopoietic stem cell transplantation in pediatric patients, demonstrating survival outcomes comparable to international series and supporting the foundation for future research.
